# World Federation for Medical Education: Appropriateness of Basic Medical Education standards in Pakistan

**DOI:** 10.12669/pjms.35.5.882

**Published:** 2019

**Authors:** Gohar Wajid, Ahsan Sethi, Rehan Ahmed Khan, Hira Shireen Aamir

**Affiliations:** 1 Dr. Gohar Wajid, MBBS, MSc, MPH, PhD Medical Education. Consultant, Health Professions Education, Institute of Health Professions Education and Research, Khyber Medical University, Peshawar, Pakistan; 2 Dr. Ahsan Sethi, BDS, MPH, MMEd, FHEA, MAcadMEd, PhD Medical Education. Assistant Professor, Health Professions Education, Institute of Health Professions Education and Research, Khyber Medical University, Peshawar, Pakistan; 3 Prof. Dr. Rehan Ahmed Khan, MBBS, FCPS, FRCS, JM-HPE, MSc-HPE. Assistant Dean Medical Education and Professor of Surgery, Riphah International University, Islamabad, Pakistan; 4 Dr. Hira Shireen Aamir, MBBS. Trainee Medical Officer, Khyber Teaching Hospital, Peshawar, Pakistan

**Keywords:** Accreditation, Basic Medical Education, Quality assurance, Standards, WFME

## Abstract

**Objective:**

To explore the appropriateness of WFME Basic Medical Education (BME) standards to Pakistani context.

**Methods:**

A descriptive cross-sectional survey of faculty, graduates and students of five Masters’ in Health Professions Education programmes was carried out from Jul-Dec 2017. Participants were asked to rate the appropriateness of WFME-BME basic standards to Pakistani context on a fourpoint Likert scale (Strongly Disagree through to Strongly Agree). They were also asked for additional comments, if any. Descriptive statistics were carried out for quantitative data, while content analysis for qualitative data.

**Results:**

A total of 127/200 participants responded (63.5%). For all major areas (106 standards), 97.7% responses (n=13,149) were positive while only 2.3% (n=313) were negative. Ninety-six standards got more than 95% positive response while only 10 standards in three major areas got more than five percent negative response. These include five standards from major area Mission and Outcomes, one from Educational Programme and four from Students.

**Conclusions:**

This is the first study exploring the appropriateness of WFME-BME standards for accreditation in Pakistan. We found that all the areas, sub-areas and standards are largely appropriate. We recommend further deliberation on 10 standards with more than five percent negative responses, regarding their contextualization to Pakistan and the development of guidelines and possible reconsiderations in WFME future revisions.

## INTRODUCTION

As the number of medical schools are increasing, the quality of medical education is drawing increasing scrutiny worldwide.^1^ Accreditation is a voluntary peer-review process intended to ensure the quality of medical education, in line with the evolving needs of the healthcare delivery system and expectations of society.^2^ Accreditation serves multiple purposes: for public, it promotes health and safety; for students, it enhances employment opportunities; for university/medical school, it provides an effective system for accountability enhancing its national and international reputation.^2^,^3^

Different government and non-government agencies perform accreditation of medical schools, using a set of standards in relation to established professional requirements, criteria and data collection tools, mostly developed through a mutual stakeholders’ consensus.^4^ For example, Liaison Committee for Medical Education in the USA^5^ and General Medical Council in UK^6^ set standards and accredit medical schools respectively. Few standards may be generic, having wider appeal, while few others may be specific to the educational context of the country.

Over the last two decades, ‘World Federation of Medical Education’ (WFME) in collaboration with the World Health Organization has worked on developing a set of standards that are predominantly generic, comprehensive, and can be adapted/adopted by countries to fit their needs.^7^ Current WFME Basic Medical Education (BME) standards^7^ are structured under nine areas with 35 sub-areas, at two levels: ‘basic standards’ or minimum requirements; and relatively advanced standards called ‘quality standards’ (Table-I).

**Table I:** WFME Basic Medical Education Standards.

Areas		Sub Areas	Standards		

			Basic	Quality	Total
1	Mission and outcomes	4	19	8	27
2	Educational programme	8	21	19	40
3	Assessment methods	2	10	5	15
4	Students	4	13	7	20
5	Academic staff and faculty	2	8	4	12
6	Educational resources	6	15	14	29
7	Programme evaluation	4	10	13	23
8	Governance and administration	5	7	8	15
9	Continuous renewal	0	3	12	5

Total		35	106	90	196

Pakistan Medical & Dental Council (PMDC) is the sole authority for accrediting, regulating and ensuring the quality of medical education in Pakistan. Existing accreditation process for medical and dental colleges at PM&DC, mainly emphasizes on the presence of infrastructure and ‘head counting’ and gives little consideration to the quality of educational processes and outcomes. PMDC plans to implement its newly developed standards through a reformed process of accreditation, and get WFME recognition.^8^ Although WFME standards are quite generic and meant to be applicable to most country situations, still, understanding the appropriateness and acceptance of these standards in a particular culture needs to be understood. This study was designed to explore the appropriateness of WFME-BME standards to Pakistani medical education context. The study also helped in identifying the standards that are less acceptable in Pakistani context and explore the reasons for their possible non-acceptance.

## METHODS

A descriptive cross-sectional survey of faculty, graduates and students of five Masters’ in Health Professions Education programmes was carried out over six months (July-December 2017). The faculty included national and international medical educationalists with postgraduate qualifications (MCPS/MS/PhD) in medical education.

### Questionnaire

A questionnaire was developed using WFME-BME basic standards^7^ as items. Four-point Likert scale (Strongly Disagree through to Strongly Agree) was used to ask participants to rate the appropriateness of each item for Basic Medical Education in Pakistan. All items were kept mandatory and optional open-ended space was added for comments. The questionnaire was validated by five experts. It was then piloted (n=10) to check for comprehension, accessibility and technical compatibility.

### Data Collection

Ethical approval was granted by Khyber Medical University Ethics Board (DIR/KMU-EB/DR/17-05 Dated:10-07-2017). A purposive sample (n=200) of faculty, students and graduates of Masters’ in Health Professions/Medical Education programmes were invited to participate in an online survey, through email. These participants were selected based on their representativeness (all geographical regions of Pakistan) and familiarity with PM&DC regulations for the recognition of medical institutes and with WFME-BME standards. Participants were informed about the implication of this research. Participation was voluntary, and participants expressed their consent by completing the questionnaire. Two reminders were sent to improve response rate.

### Data Analysis:

The quantitative data from the questionnaires were coded and entered into SPSS.v.24. Descriptive statistics (frequencies and percentages) were calculated, stratified by Area, Sub-Area and Standard. Responses were grouped into positive responses (Strongly Agree and Agree) and negative responses (Strongly Disagree and Disagree) of that Standard/Area/Sub Area. A content analysis was carried out on the qualitative data.^9^

## RESULTS

A total of 127 participants responded to the survey (response rate=63.5%). There was almost equal participation from both genders, with age ranging from 26-65 years. Most respondents were actively involved in teaching (n=110) and working against a faculty position (n=93). Many had MBBS/BDS as basic qualification along with clinical/basic sciences postgraduate qualifications before acquiring MME/MHPE/MCPS in medical education. A majority (n=116) reported having prior familiarity with WFME standards (Table-II).

**Table II:** Participant characteristics.

Characteristics			Frequency	Percentage
Gender	Male		67	52.8
	Female		60	47.2
Age	26-35 Yrs.		29	22.8
	36-45 Yrs.		48	37.8
	46-55 Yrs.		34	26.8
	56-65 Yrs.		16	12.6
Medical Education Qualification	MME/MHPE Student		15	11.8
	MME/MHPE/MCPS		108	85.0
	PhD Medical Education		4	3.1
Level of Familiarity with WFME Standards	Heard but never read before		11	8.7
	Familiar		73	57.5
	Very Familiar		43	33.9
Faculty Type	Basic Sciences		58	45.7
	Clinical Sciences		52	40.9
	Not Teaching Currently		17	13.4
Rank	Professor		28	22.0
	Associate Professor		23	18.1
	Assistant Professor		42	33.1
	Lecturer/Registrar		24	18.9
	Not Applicable		10	7.9
Teaching Experience	More than 10 Yrs.		50	39.4
	6-10 Yrs.		22	17.3
	1-5 Yrs.		46	36.2
	Not Applicable		9	7.1
Workplace	Punjab		58	45.7
	Khyber Pakhtunkhwa		34	26.8
	Sindh		9	7.1
	Baluchistan		4	3.1
	Azad Jammu Kashmir		6	4.7
	Federal		8	6.3
	International		8	6.3

The distribution of participants’ responses on nine major areas is shown in Table-III. For all major areas (106 standards), an overwhelming majority of responses (97.7%) were positive while only 2.3% were negative. Negative responses varied between 1% as minimum for major area eight (Governance and Administration) to 4.4% as maximum for major areas one (Mission and Outcomes) and four (Students). Of all standards, 96 got more than 95% positive response, while only 10 standards got negative response by more than 5% respondents. These include five from major area one (Mission and Outcomes), one from major area two (Educational Programme) and four from major area four (Students).

**Table III:** Summary of positive and negative responses on basic standards.

Major areas	Number of Basic Standards	Strongly Agree and Agree (Positive response)		Strongly Disagree and Disagree (Negative response)		Standards with more than 5% negative response

		N	%	N	%	
1: Mission and Outcomes	19	2307	95.6	106	4.4	B116, B121, B122, B134, B141
2: Educational Programme	21	2621	98.3	46	1.7	B242
3: Assessment methods	10	1253	98.7	17	1.3	Nil
4: Students	13	1578	95.6	73	4.4	B441, B442, B443, B444
5: Academic Staff/Faculty	8	1005	98.9	11	1.1	Nil
6: Educational Resources	15	1880	98.7	25	1.3	Nil
7: Programme Evaluation	10	1250	98.4	20	1.6	Nil
8: Governance and Administration	7	880	99.0	9	1.0	Nil
9: Continuous Renewal	3	375	98.4	6	1.6	Nil
Grand total for all standards	106	13149	97.7	313	2.3	Nil

**Fig.1:**
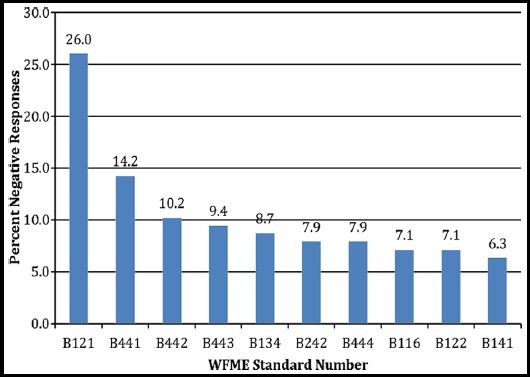
WFME-BME standards with more than five percent negative responses.

WFME-BME Standards with more than 5% negative responses are shown in Fig-1. Standard B1.2.1, on appropriateness of giving institutional autonomy to medical schools in formulation and implementation of the curriculum received maximum negative responses (26%). Some respondents believed that PM&DC should develop the curriculum: *‘there should be a uniform curriculum’* or provide guidelines: *‘governing body must play a key role in providing guideline’* because institutional autonomy might result in varying levels of competence among graduates from different institutions: *‘institutional autonomy…can result in large variation among institutions and can effect students learning’*. Others suggested: *‘Institutions may be permitted to make some adjustments with approval’* over and above a baseline curriculum.

Few participants also disagreed on allowing student representation and participation in developing mission statement (B-4.4.1, 4.2%), design (B-4.4.2, 10.2%), management (B-4.4.3, 9.4%) and evaluation (B-4.4.4, 7.9%) of the programme. They were of the opinion: *‘students are not aware of the management process and administrative work…so their participation is not likely to be effective’*, *‘Students involvement should be at a limited level…they make immature suggestions…biased comments’*. Others believed that students’ representation is important, but they need to be trained: *‘a long and strenuous effort of a change in student culture is required before they can be given an effective representation’*.

Some recommended that the mission statements should not incorporate reference to postgraduate medical education (B-1.1.6, 7.1%) at undergraduate level: *‘mission should be crisp and brief so that everyone is able to memorize it. If we use too many variables it will really become a story and will not serve the purpose’*. They were also not in favor of medical schools having undergraduate educational outcomes in relation to their subsequent postgraduate training (B-1.3.4, 8.7%). They opined: *‘the focus should be basic medical education otherwise the students are more concerned about postgraduate roles’*.

Few were not in favor of having social sciences in the core curriculum (B-2.4.2, 7.9%) and suggested that it can be introduced as an elective for those interested. Some participants were also against giving institutional autonomy regarding the use of resources (B-1.2.2, 7.1%) as they feared unjust use.

A minority also disapproved the involvement of all stakeholders in formulating the mission and outcomes (B-1.4.1, 6.3%). They believed that it would be: ‘*very difficult to get all the principal stakeholders to agree upon a set of values’*. Others felt: *‘Educationist alone should formulate the mission and intended educational outcomes’*.

## DISCUSSION

The study explored the perceptions of medical educators on the appropriateness of WFME-BME standards to Pakistani medical education context. Even though, the interpretation of WFME standards and their use in evaluation has been reported as challenging^1^, ninety-six standards received more than 95% positive response, while only ten standards received a negative response by more than five percent respondents. This endorses the claim by WFME that these standards are generic and meant to be applicable to most country situations.^2^,^7^ Some countries including Ireland and Australia have already adapted WFME standards for accreditation purpose.^10^ PMDC recently developed a new set of standards.^11^ Though the ‘inspection’ function has been renamed as ‘accreditation’, the transformation of the function from inspection to accreditation has not fully completed yet. The new standards are yet to be tested for their validity, measurability, acceptability and compatibility with both the local context and changing global scenario. The findings from this study may provide guidelines for further contextualization of PM&DC standards and ensure the best use of resources.

Standards with more than five percent negative response included five from major area one (Mission and Outcomes), one from major area two (Educational Programme) and four from major area four (Students). An in-depth understanding of the negative responses may help inform accreditation standards and subsequently instruments that address local needs of the community and are globally acceptable as well.^2^ As the major purpose of WFME standards is to encourage self-evaluation and bring improvements among institutions providing medical education^10^, these standards would need further deliberation in WFME future revisions to ensure their wider applicability in countries having similar educational context.

Twenty-six percent of the participants disagreed on giving autonomy to medical schools in formulation and implementation of the curriculum. Traditionally, the PM&DC has been providing syllabus for medical education to the colleges.^12^ This prescribes minimum requirements for the content and duration allocated to each subject, and a uniform criterion for assessment. Different competencies for a medical doctor have been proposed to meet the needs of the community, which requires knowledge and skills beyond traditional teaching of basic and clinical subjects.^13^ At the same time, the increased public expectations, accreditation requirements and evidence-based education have resulted in professionalization of medical education.^14^,^15^ As institutions have different resources, therefore, a straitjacket approach towards curriculum may not be feasible. Recognizing the participants’ fears about the misuse of institutional autonomy for curriculum development, leading to misalignment between the kinds of doctors produced and the ones actually needed in Pakistan, PM&DC needs to issue curriculum development guidelines that clearly demarcate the role and responsibilities of medical schools in developing their curricula. Several such guidelines for example Tomorrows Doctor^16^ and CanMEDS physician competency framework^17^ are already available. A careful evaluation of medical schools’ capacity to develop their own curricula, backed by robust training of medical education department to take a lead in developing such curricula is also recommended.

Many participants thought student representation and participation in curriculum development, management and evaluation as inappropriate. As consumers of institutional services, students are perhaps the most important stakeholder group in higher education.^18^ The institutions must engage students in the management, delivery and evaluation of their services. Students should be consulted, given certain rights and responsibilities in all academic matters that concern them. Nowadays, many institutions are encouraging students to get actively involved in their medical education, who are also keen to learn and contribute.^19^

Few participants in the current study were not in favor of giving autonomy in the use of resources as they feared their unjust use and nepotism. Private-sector institutions in Pakistan enjoy more administrative and financial autonomy compared with public-sector institutes, at times resulting in wide variations in staff salaries and subsequently progression. Realizing the situation, provincial governments have started to fill the gaps. For example, the government of the province of Khyber Pakhtunkhwa has introduced Medical Teaching Institutions reforms to enhance administrative and financial autonomy of medical institutions, leading to market-based salaries/incentives for the faculty.^20^

### Limitations of the study

Comparison of all aspects of the results was difficult as our literature search did not identify similar studies conducted on WFME standards. This study may not be generalizable as there was less representation of participants from the province of Sindh (7%). Despite the limitations, the findings provide useful insights to begin an explicit discussion concerning the appropriateness of WFME standards.

## CONCLUSION

This is the first study exploring the appropriateness of WFME-BME standards for accreditation in Pakistani medical education context. We found that all the areas, sub-areas and standards are largely appropriate. Only 10 standards, five from major area one (Mission and Outcomes), one from major area two (Educational Programme) and four from major area four (Students) provoked reservations. We recommend further deliberation on these standards, regarding their contextualization to Pakistan and the development of guidelines, and possible reconsiderations in WFME future revisions. Future research needs to explore the challenges towards accreditation qualitatively.
